# VEGF-A Immunohistochemical Expression Across Appendiceal Epithelial Lesions: A Retrospective Exploratory Study

**DOI:** 10.3390/medsci14060684

**Published:** 2026-10-19

**Authors:** Ante Tavra, Marina Ćaleta Rade, Toni Čeprnja, Bernarda Lozić, Marin Ogorevc, Jure Krstulović, Zrinka Hrgović, Ante Krešo, Ognjen Barčot

**Affiliations:** 1 Department of Paediatrics, Section of Medical Genetics, University Hospital of Split, Spinčićeva 1, 21000 Split, Croatia; 2 School of Medicine, University of Split, 21000 Split, Croatia; jure.krstulovic@mefst.hr (J.K.); zrinkahrgovic@hotmail.com (Z.H.);; 3 Department of Pathology, Judicial Medicine, and Cytology, University Hospital of Split, 21000 Split, Croatia; 4 Division of Abdominal Surgery, Department of Surgery, University Hospital of Split, 21000 Split, Croatia; 5 Department of Family Medicine, Health Center of Split-Dalmatia County, 21000 Split, Croatia; 6 Department of Ophthalmology, University Hospital of Split, 21000 Split, Croatia

**Keywords:** appendiceal neoplasm, low-grade appendiceal mucinous neoplasm, VEGF-A, angiogenesis, sessile serrated lesion, immunohistochemistry

## Abstract

**Background:** Appendiceal epithelial lesions are rare and heterogeneous. Evidence regarding vascular endothelial growth factor A (VEGF-A) expression in localized appendiceal lesions is limited. We compared VEGF-A immunoreactivity across diagnostic subtypes and explored associations with histological features. **Methods:** This retrospective exploratory study included 73 appendiceal neoplastic epithelial lesions. Formalin-fixed, paraffin-embedded tissue was assessed for predominant VEGF-A staining intensity and percentage of positive neoplastic epithelial cells. Their product was reported as a modified immunoreactivity score (0–300). Permutation-based nonparametric tests, Benjamini–Hochberg correction, diagnosis-adjusted sensitivity analyses, and interobserver agreement analyses were used. **Results:** VEGF-A staining was present in 71/73 cases. Median immunoreactivity scores were 205 for adenocarcinoma, 170 for hyperplastic polyp, 180 for low-grade appendiceal mucinous neoplasm, and 100 for sessile serrated lesion; the overall difference was not significant (*p* = 0.073). No histological-feature association remained significant after false-discovery-rate correction. Surface nuclear stratification was not significant in the global analysis (*p* = 0.051; q = 0.169), and after diagnosis adjustment (*p* = 0.282). VEGF-A measures did not differ between acute and incidental presentations; patients with acute presentations were younger (*p* = 0.006). **Conclusions:** VEGF-A immunoreactivity was common, but no robust subtype-specific or histology-specific pattern was identified. These exploratory findings do not establish clinical utility and require confirmation in larger controlled cohorts.

## 1. Introduction

Epithelial lesions of the vermiform appendix are rare and encompass biologically distinct entities, including hyperplastic polyps, sessile serrated lesions (SSLs), low-grade appendiceal mucinous neoplasms (LAMNs), and adenocarcinomas [1,2,3,4]. Their clinical presentation is often nonspecific: lesions may be discovered after appendectomy for acute appendicitis, incidentally during another abdominal procedure, or during investigation of disseminated mucinous disease [5,6].

LAMN is characterized by low-grade mucinous epithelium and a distinctive pattern of mural involvement, with a risk of peritoneal dissemination when neoplastic epithelium or mucin extends beyond the appendix [1,2,3,4]. Current classification systems emphasize accurate distinction among localized epithelial lesions because their biological behavior and management differ substantially [2,3,4].

Angiogenesis supports tumor growth and progression by supplying oxygen and nutrients and by modifying the tumor microenvironment [7,8]. Vascular endothelial growth factor A (VEGF-A) is a principal ligand in this process and acts mainly through VEGF receptors, particularly VEGFR-2 [9,10,11,12]. Hypoxia and inflammation can also induce VEGF-A signaling, while VEGF-A may influence antitumor immune responses [13,14].

Because VEGF-A, total VEGF immunoreactivity, and VEGF-receptor expression represent related but non-equivalent measurements, findings from different assays and disease settings should not be interpreted interchangeably [9,10,11,12]. This distinction is especially important in rare appendiceal tumors, for which available biomarker evidence is limited.

The most directly relevant appendiceal studies examined advanced disease. VEGF immunoreactivity was associated with survival in a small cohort dominated by mucinous appendiceal adenocarcinoma with peritoneal metastases, whereas a separate study evaluated VEGFR-2 rather than VEGF-A in appendiceal carcinomatosis [15,16]. Neither study characterized VEGF-A across the spectrum of localized appendiceal epithelial lesions.

Studies of colorectal neoplasia demonstrate that VEGF-related pathways can be detected in carcinomas and polyps, but anatomical site, lesion classification, antibodies, scoring systems, and comparator tissues differ among reports [17,18]. These data provide biological context but not a direct reference standard for appendiceal lesions.

We therefore aimed to estimate VEGF-A immunohistochemical expression across localized appendiceal neoplastic epithelial lesion subtypes and to explore its associations with selected architectural, cytological, mucin-related, inflammatory, and proliferative features. The study was designed as a retrospective exploratory analysis rather than as a validation of a diagnostic, prognostic, or predictive biomarker.

## 2. Patients and Methods

### 2.1. Study Design

This retrospective observational study investigated the immunohistochemical expression of VEGF-A in appendiceal neoplasms and its association with histopathological characteristics. The study was conducted at the Department of Pathology and the Department of Surgery, University Hospital of Split, Croatia.

Archived histopathological reports and corresponding formalin-fixed, paraffin-embedded (FFPE) tissue specimens from patients who underwent surgical treatment between 1 January 2017 and 31 December 2021 were reviewed. This study period was selected to ensure uniformity of institutional histopathological processing procedures, availability of archived tissue blocks, and completeness of clinical and pathological documentation. Clinical management and surgical treatment were determined according to standard institutional practice and were not influenced by study participation. Cases were identified retrospectively through the institutional pathology database.

Eligible patients were adults (≥18 years) with histologically confirmed appendiceal neoplasms and available FFPE tissue blocks suitable for immunohistochemical analysis. Patients were identified from three clinical settings: (1) appendectomy performed for acute appendicitis, (2) appendiceal specimens incidentally removed during surgery for unrelated primary abdominal pathology, and (3) right hemicolectomy specimens containing the appendix. Acute appendectomies were classified as acute presentations; incidental removals and all elective right hemicolectomies were classified as incidental presentations. Patients were excluded if they were younger than 18 years, lacked a definitive histopathological diagnosis, had incomplete accompanying pathology documentation, had insufficient or unavailable FFPE tissue material, or had an indeterminate diagnosis following histopathological review.

Archived FFPE tissue blocks obtained during routine diagnostic processing were used for immunohistochemical analysis. Following surgical excision, specimens were fixed in 10% neutral buffered formalin (for 12–24 h, exceptionally up to 72 h) and processed according to institutional pathology protocols before paraffin embedding and long-term storage in the departmental tissue archive. Representative tumor sections were selected by a gastrointestinal pathologist based on hematoxylin and eosin (H&E)-stained slides.

All diagnoses were classified according to the 2019 WHO Classification of Tumours of the Digestive System [4]. The cohort included four adenocarcinomas (one goblet cell adenocarcinoma and three adenocarcinomas not otherwise specified), nine HPs, 17 LAMNs, and 43 SSLs. Among the SSLs, 31 were without dysplasia and 12 had low-grade dysplasia; no SSL with high-grade dysplasia was identified. SSLs with and without low-grade dysplasia were pooled for the prespecified exploratory analyses because histological components were evaluated across the lesion spectrum independently of diagnostic subtype.

All four adenocarcinomas invaded the subserosa or mesoappendix without penetration of the serosal surface and were classified as pT3. One to two regional lymph nodes were available per case; all were negative, supporting pN0, although the nodal yield was limited. Pathological distant-metastasis status was not assigned. MSCT of the thorax and MRI of the abdomen and pelvis showed no radiologically detectable extra-appendiceal, peritoneal, or distant disease.

In all 17 LAMNs, disease remained without signs of extra-appendiceal disease; no acellular mucin or neoplastic epithelium was identified in the subserosa, mesoappendix, or serosal surface. No perforation or extra-appendiceal mucin was recorded, and resection margins were negative.

The primary objective was to determine VEGF-A expression in appendiceal neoplastic columnar epithelial cells using immunohistochemistry and to evaluate its association with histological features. Secondary objectives included comparison of VEGF-A expression among appendiceal neoplasm subtypes and assessment of VEGF-A expression across individual histopathological components irrespective of tumor subtype.

The study was approved by the institutional ethics board of the University Hospital of Split, Croatia (29 January 2024; approval no. 520-03/24-01/12) and was reported in accordance with the REMARK (Reporting Recommendations for Tumor Marker Prognostic Studies) guidelines [19].

### 2.2. Data Collection

Clinical and pathological data were extracted retrospectively from archived pathology reports and associated patient records. The following variables were collected: age, sex, original histopathological diagnosis, and histopathological features of interest. The histopathological characteristics examined were: histological subtype of appendiceal neoplasm, overall architectural pattern, crypt serration, basal crypt dilatation, nuclear stratification within crypts, nuclear stratification on the epithelial surface, severity of inflammatory infiltrate, distribution of inflammatory infiltrate, presence of vesicular mucin, presence of luminal mucin, degree of nuclear atypia, and mitotic count per mm^2^ (400× magnification).

Histological features were assessed on H&E-stained sections using operational categories adapted from the semiquantitative morphological framework of Torlakovic et al. [20]. Overall architectural distortion denoted loss of the normal appendiceal lymphoid tissue and straight tubular crypt pattern, replaced by flattened, undulating, villous, back-to-back, branching, cribriform, or finger-like mucosal architecture. HP was distinguished by serrated epithelium without unequivocal basal crypt distortion, whereas SSL required at least one unequivocally distorted serrated crypt showing basal dilatation, horizontal growth along the muscularis mucosae, serration extending to the crypt base, asymmetric proliferation, or boot-shaped/branching architecture. LAMN required low-grade mucinous epithelium with characteristic mural involvement and excluded infiltrative destructive invasion [1,2,3,4].

Crypt dilatation was defined as abnormal widening, expansion, or cystic enlargement of crypt lumina and was characterized as basal when involving the deepest third, diffuse when involving the full crypt length, or luminal when predominating in the upper half. Nuclear stratification was abnormal when nuclei lost basal polarity and extended beyond the basal half into the upper third or luminal surface. Nuclear atypia incorporated increased nuclear-to-cytoplasmic ratio, hyperchromasia, pleomorphism, coarse chromatin, and atypical mitoses. Vesicular and luminal mucin were graded semiquantitatively as none, mild, moderate, or severe; extracellular mucin dissecting through muscularis mucosae defects was evaluated separately in the diagnostic assessment of LAMN.

Inflammatory-infiltrate severity was categorized as none, mild (<10 scattered inflammatory cells/HPF), moderate (small clusters of 10–50 cells/HPF), or severe (dense sheets > 50 cells/HPF). Distribution was recorded as focal, interstitial, or diffuse and was left blank/not applicable when no inflammatory infiltrate was present.

Mitoses were counted on H&E sections in five non-overlapping high-power fields, each corresponding to 0.20 mm^2^, yielding a total assessed area of 1.0 mm^2^ at 400× total magnification.

All data were anonymized before statistical analysis and entered into a dedicated database using Microsoft Excel 2010 (Microsoft Corporation, Redmond, WA, USA).

### 2.3. Immunohistochemistry

VEGF-A immunohistochemistry was performed on FFPE sections using a standardized laboratory protocol under uniform conditions. Placental tissue served as an external positive tissue control. No separate no-primary reagent control was performed. Non-lesional stromal tissue present within the study sections was used only as an internal negative tissue. The four adenocarcinomas were analyzed as study cases, not as technical controls; their expected reactivity served only as an additional biological face-validity check. Full sectioning, antigen-retrieval, antibody, detection, quality-control, and reproducibility details are provided in Supplementary File S1.

VEGF-A immunoreactivity was defined as brown cytoplasmic staining in neoplastic columnar epithelial cells. Predominant staining intensity was scored as 0 (absent), 1 (weak), 2 (moderate), or 3 (strong), and the percentage of positive neoplastic cells was estimated from 0% to 100%. A modified immunoreactivity score was calculated as predominant intensity multiplied by the percentage of positive cells, yielding a range of 0–300.

The entire lesional area was first reviewed at low magnification to assess the overall distribution of staining. Five non-overlapping representative fields were then selected across the lesion, avoiding selection based solely on staining strength. The percentage of VEGF-A-positive neoplastic columnar epithelial cells was visually estimated across the assessed fields in 5% increments from 0% to 100%. Predominant staining intensity was defined as the intensity category (0–3) representing the greatest proportion of stained lesional epithelial cells across the assessed area, rather than the maximum focal intensity observed. Only lesional columnar epithelial cells were scored. Stromal and inflammatory cells were excluded from the percentage and intensity assessments.

This product is not the conventional weighted H-score, which sums the proportions of cells at each intensity. It is therefore termed the modified immunoreactivity score throughout. Values stored in the source spreadsheet on a 0–3 scale were multiplied by 100 for reporting on the 0–300 scale; this linear rescaling does not alter rank-based or analysis-of-variance *p*-values. Similar modified H-score approaches, based on multiplication of staining intensity and the percentage of positive cells, have been used in multiple previous immunohistochemical studies across different tumor types [21,22,23].

Two experienced gastrointestinal pathologists scored intensity and percentage positivity independently while blinded to clinical data and to the recorded diagnostic label; morphological features visible on the slide could not be masked. Slides were coded before assessment. Discrepancies were resolved by joint review and consensus, with a third pathologist consulted when needed. Consensus values were used for primary analyses.

### 2.4. Statistical Analysis

The modified immunoreactivity score was the primary VEGF-A outcome; predominant intensity and percentage positivity were secondary. Mitotic activity and associations with ten histological features were exploratory. Continuous and ordinal variables were summarized using medians and interquartile ranges (IQRs), with means and standard deviations where informative; categorical variables were summarized as counts and percentages.

Two-group comparisons used Wilcoxon rank-sum statistics, and comparisons across three or more categories used tie-corrected Kruskal–Wallis H statistics based on midranks. *p*-values were two-sided for rank-sum tests and upper-tail for H, Pearson chi-square, and F statistics. Monte Carlo *p*-values were calculated as c/n, where c was the number of permuted statistics at least as extreme as observed and n was the number of permutations. Analyses used 99,999 permutations and were repeated with 999,999 permutations when the Monte Carlo 95% confidence interval crossed 0.05. The base seed was 20261007; test-specific seeds were generated deterministically by adding the sequential test number.

Epsilon-squared was reported for Kruskal–Wallis comparisons. For the primary diagnostic comparison of the modified score, a diagnosis-stratified percentile-bootstrap 95% confidence interval used 20,000 resamples. Pairwise rank-sum comparisons used Holm adjustment. Benjamini–Hochberg false-discovery-rate correction was applied separately across the ten-feature tests within each outcome family, with q < 0.05 as the multiplicity-adjusted threshold.

Interobserver agreement was assessed before consensus scoring using linear-weighted Cohen’s kappa for ordinal intensity and ICC(A,1) for percentage positivity and the modified score. The 95% confidence interval for kappa used 20,000 percentile-bootstrap resamples with seed 20261007; ICC confidence intervals were model-based.

Sparse intensity-by-feature tables were evaluated using fixed-margin Monte Carlo permutation of the Pearson chi-square statistic rather than asymptotic chi-square inference. Monte Carlo 95% confidence intervals were calculated for permutation *p*-values.

Diagnosis-adjusted sensitivity analyses used Freedman-Lane residual permutation on outcome midranks: reduced models contained diagnostic subtype and full models added the feature of interest. The modified score-mitosis association used Spearman’s rank correlation. Acute-versus-incidental comparisons used the same permutation framework; the modified-score sensitivity model additionally adjusted for diagnosis and age. Missing data were handled by complete-case analysis (n = 72 for nuclear atypia; n = 59 for inflammatory-infiltrate distribution after excluding not-applicable cases). Non-estimable or rank-deficient terms were not interpreted. Unless otherwise specified, *p* < 0.05 and, for corrected families, q < 0.05 were the prespecified thresholds.

All analyses were performed in Stata 14.0 (StataCorp, College Station, TX, USA).

## 3. Results

### 3.1. Patient Characteristics and VEGF-A Staining in Appendiceal Neoplasms

**Figure 1:**
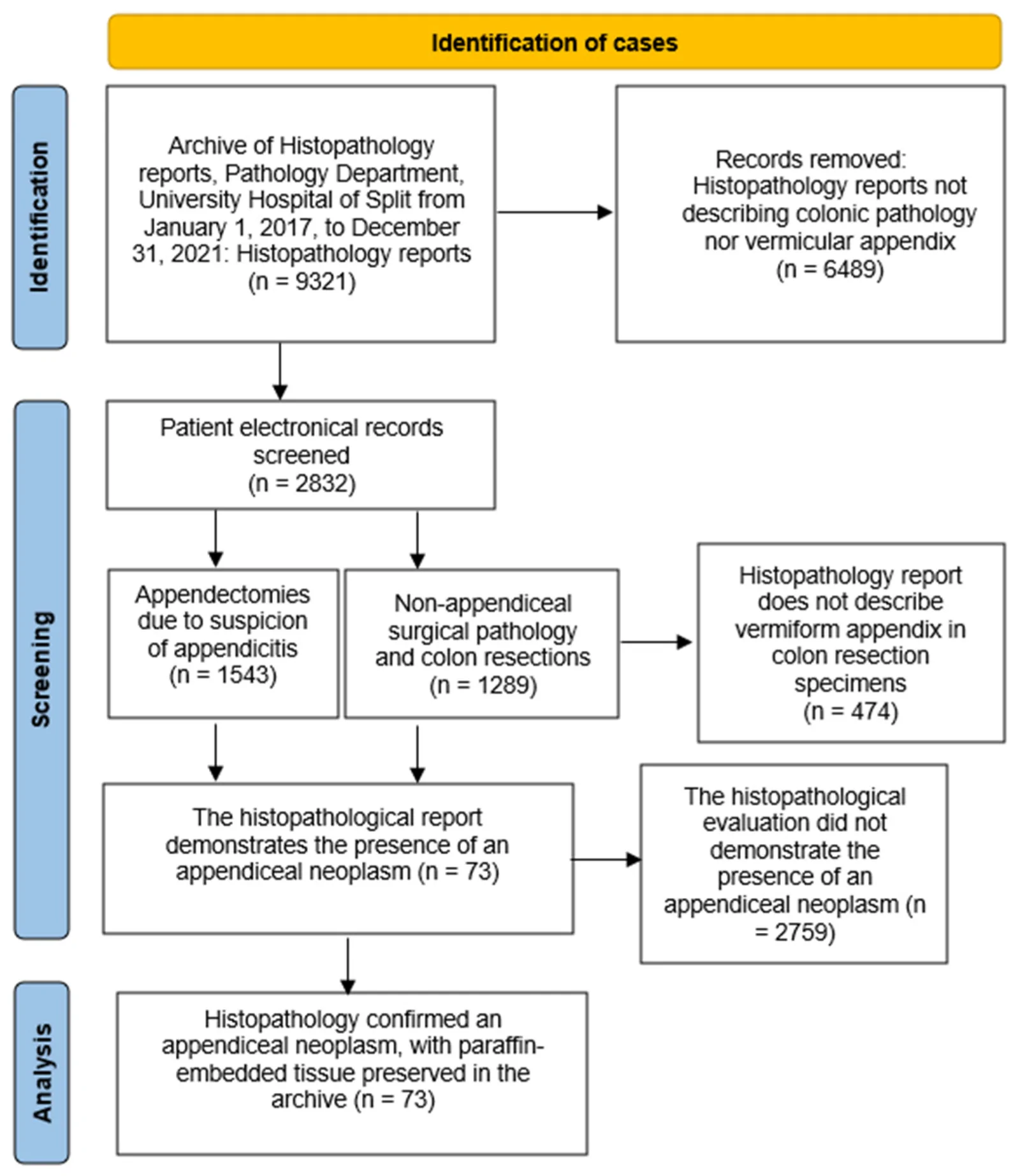
Flow diagram of case selection.

A total of 2832 pathology reports were screened. Of these, 2759 did not meet the inclusion criteria, including 474 colonic resection reports in which the appendix was not described or not included in the histopathological evaluation. Seventy-three cases had an eligible appendiceal epithelial lesion and available FFPE tissue (Figure 1). Thirty-six (49.3%) were acute presentations and 37 (50.7%) were incidental. The cohort comprised four adenocarcinomas, nine hyperplastic polyps, 17 LAMNs, and 43 SSLs. Baseline characteristics by diagnosis are shown in Table 1.

**Figure 2:**
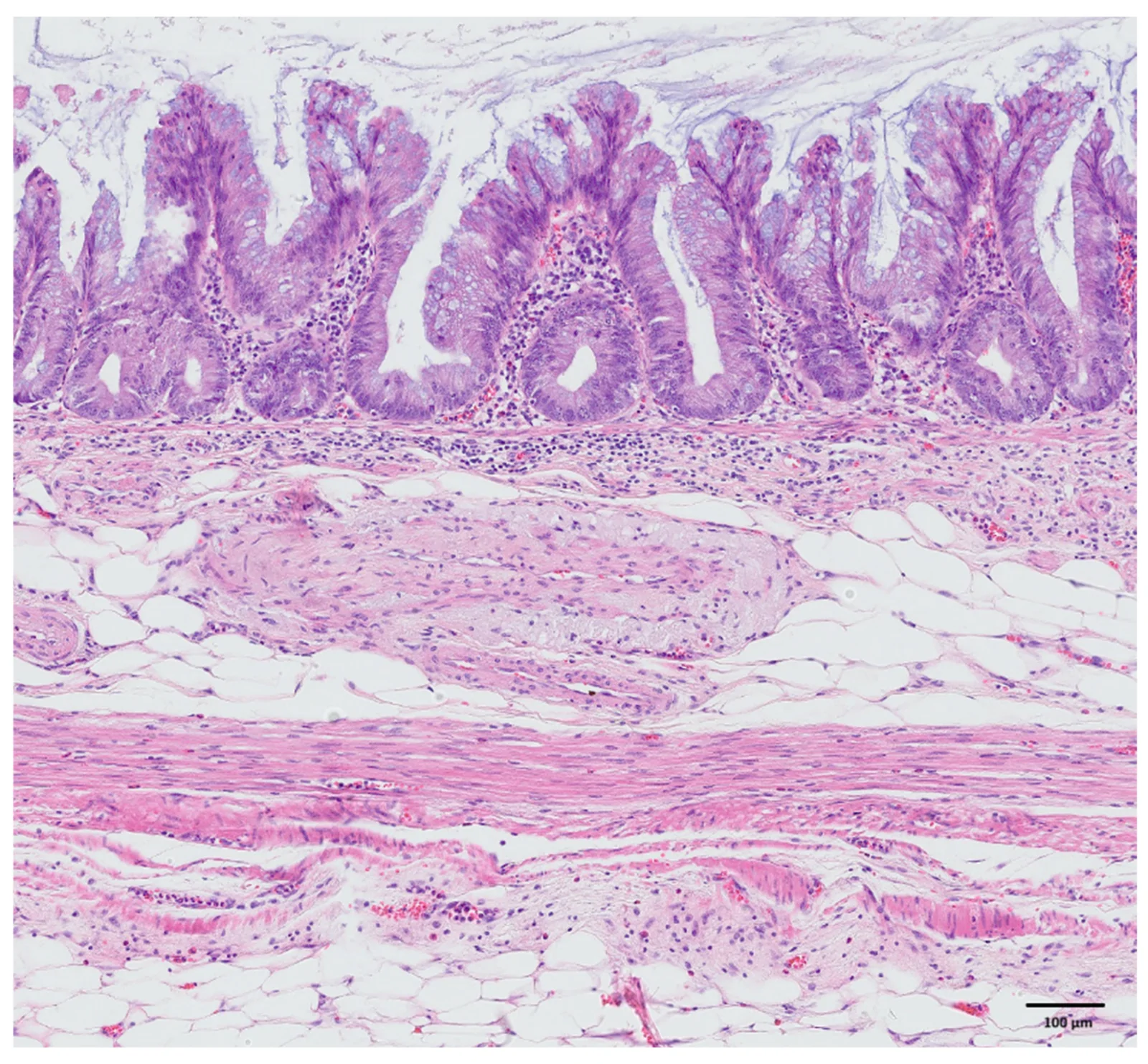
Low-grade appendiceal mucinous neoplasm (LAMN) with representative hematoxylin and eosin (H&E) staining. Scale bar = 100 µm.

**Figure 3:**
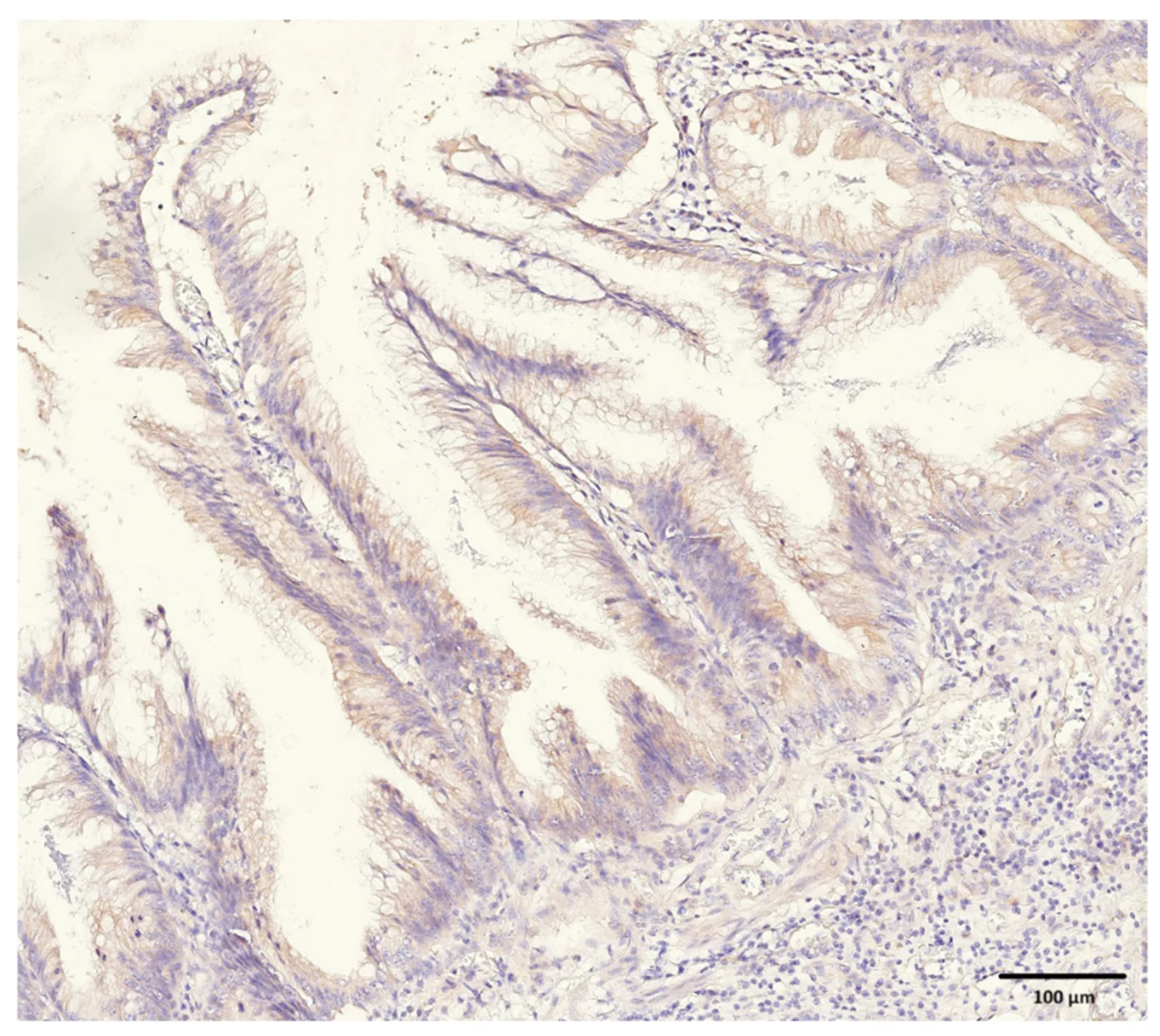
Low-grade appendiceal mucinous neoplasm (LAMN) with strong VEGF-A cytoplasmic immunoreactivity. Scale bar = 100 µm.

**Figure 4:**
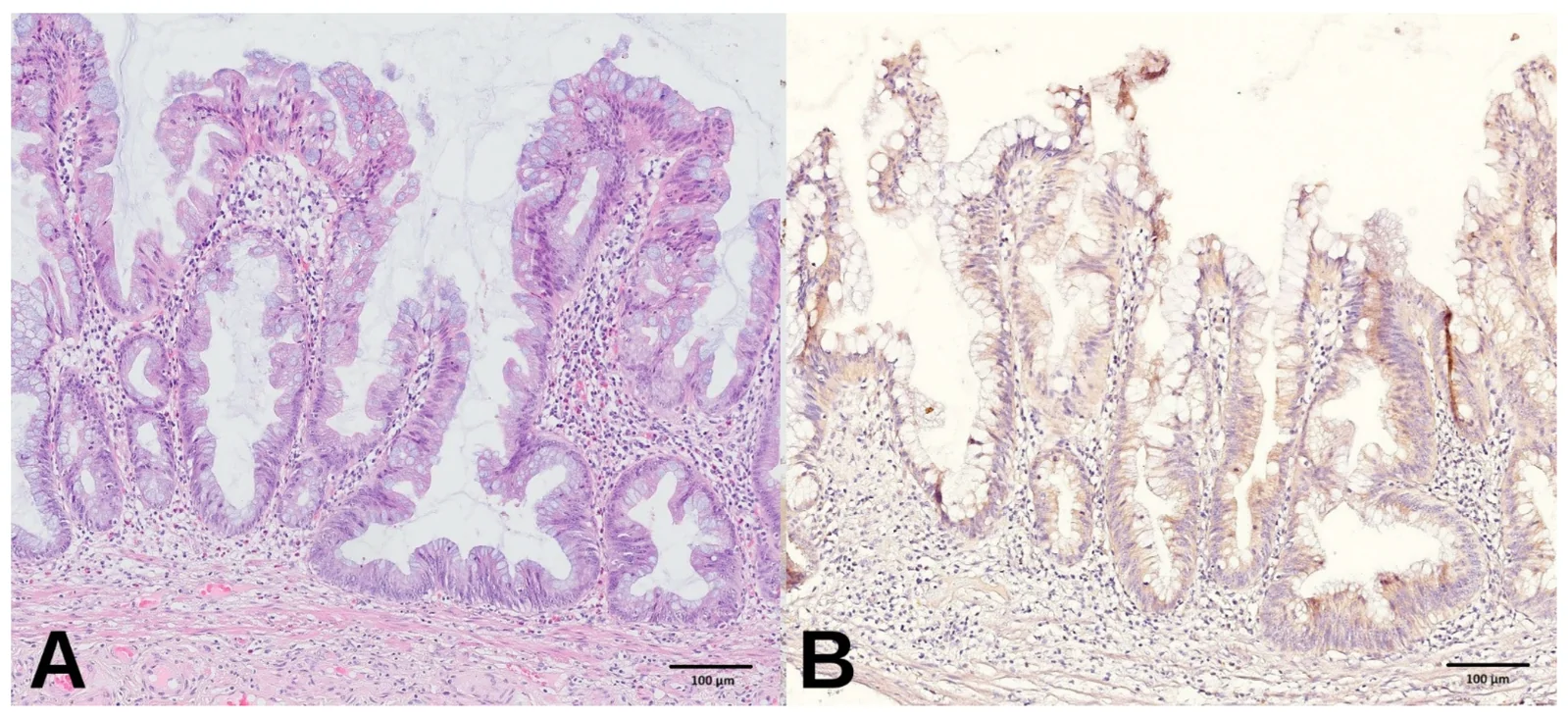
Representative histopathological features of a sessile serrated lesion (SSL): (**A**) hematoxylin and eosin (H&E) staining and (**B**) VEGF-A immunohistochemistry showing strong cytoplasmic immunoreactivity. From the same specimen. Scale bars = 100 µm.

**Figure 5:**
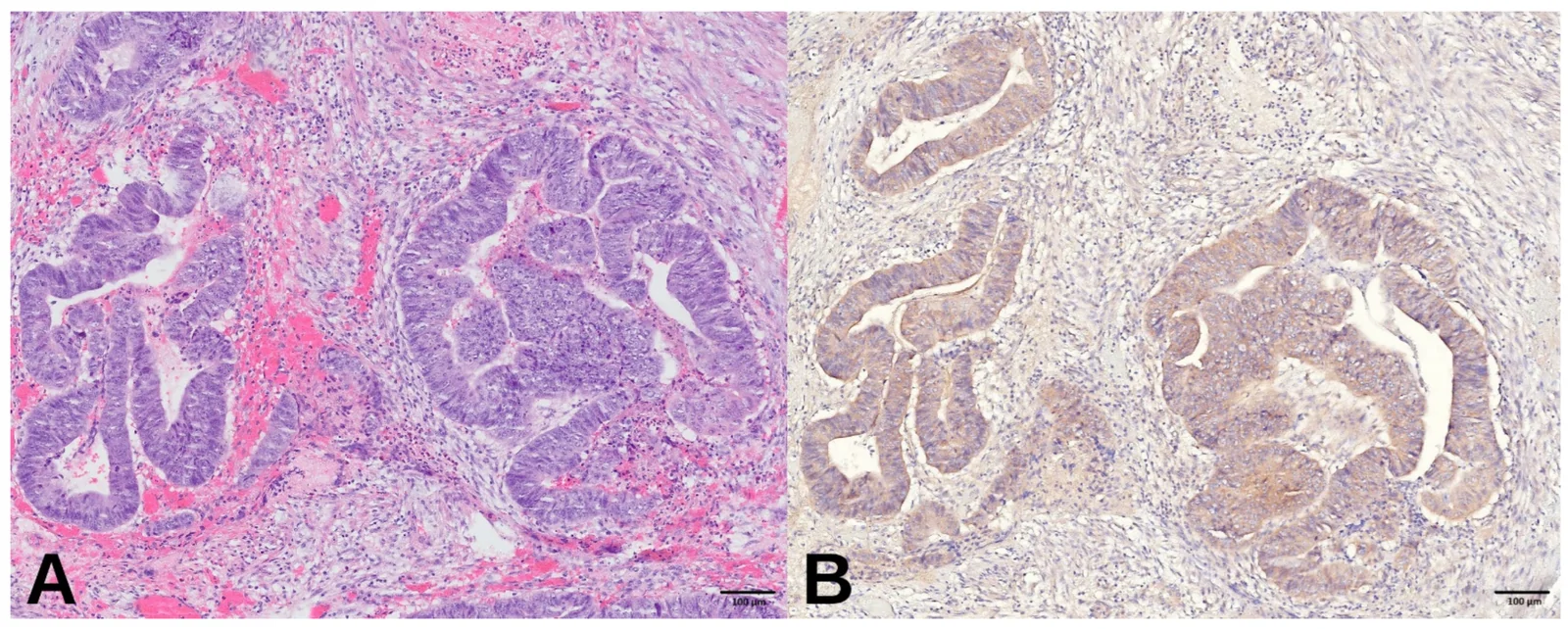
Representative histopathological features of appendiceal adenocarcinoma (ADCA): (**A**) hematoxylin and eosin (H&E) staining and (**B**) VEGF-A immunohistochemistry showing strong cytoplasmic immunoreactivity. From the same specimen. Scale bars = 100 µm.

**Figure 6:**
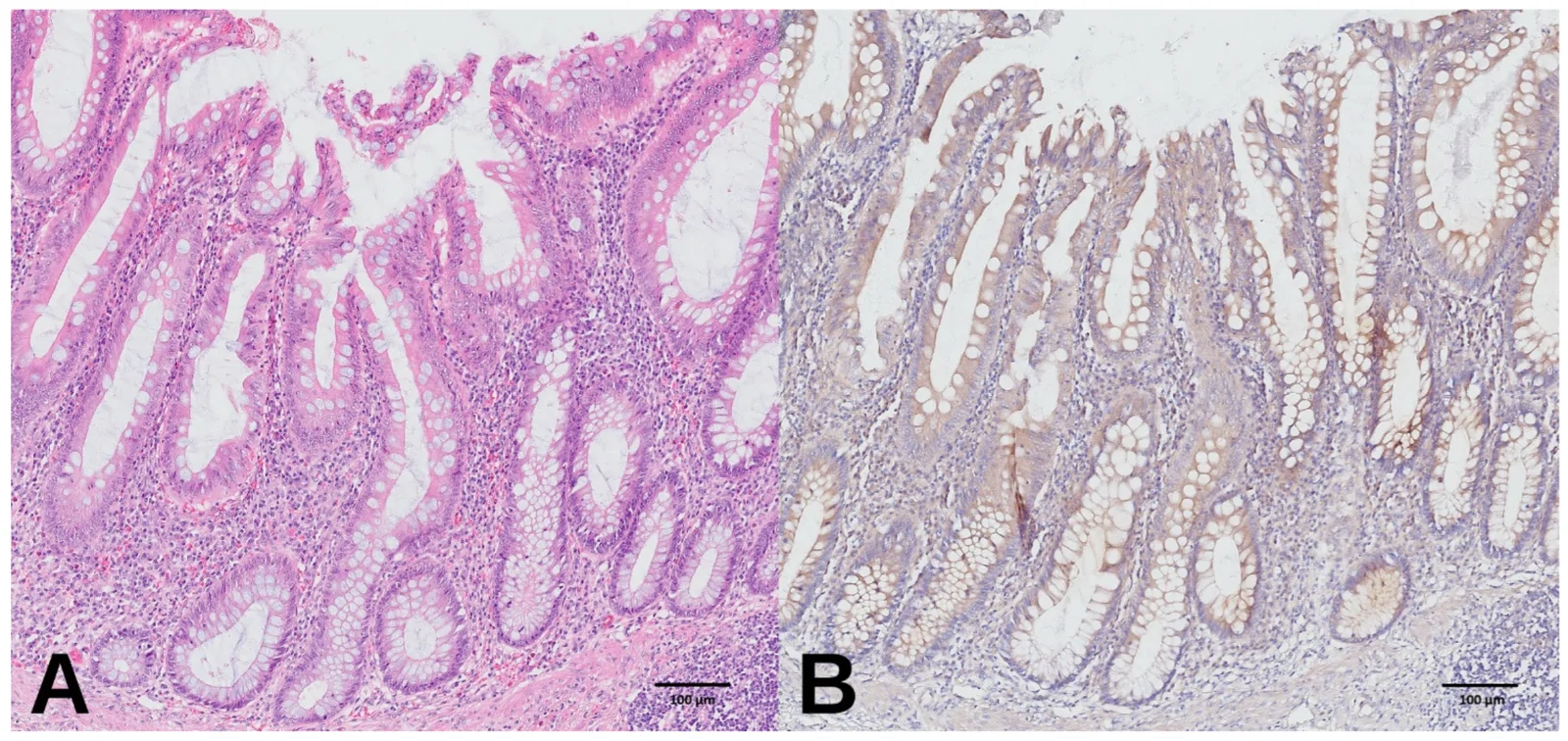
Representative histopathological features of an appendiceal hyperplastic polyp (HP): (**A**) hematoxylin and eosin (H&E) staining and (**B**) VEGF-A immunohistochemistry showing strong cytoplasmic immunoreactivity. From the same specimen. Scale bars = 100 µm.

**Figure 7:**
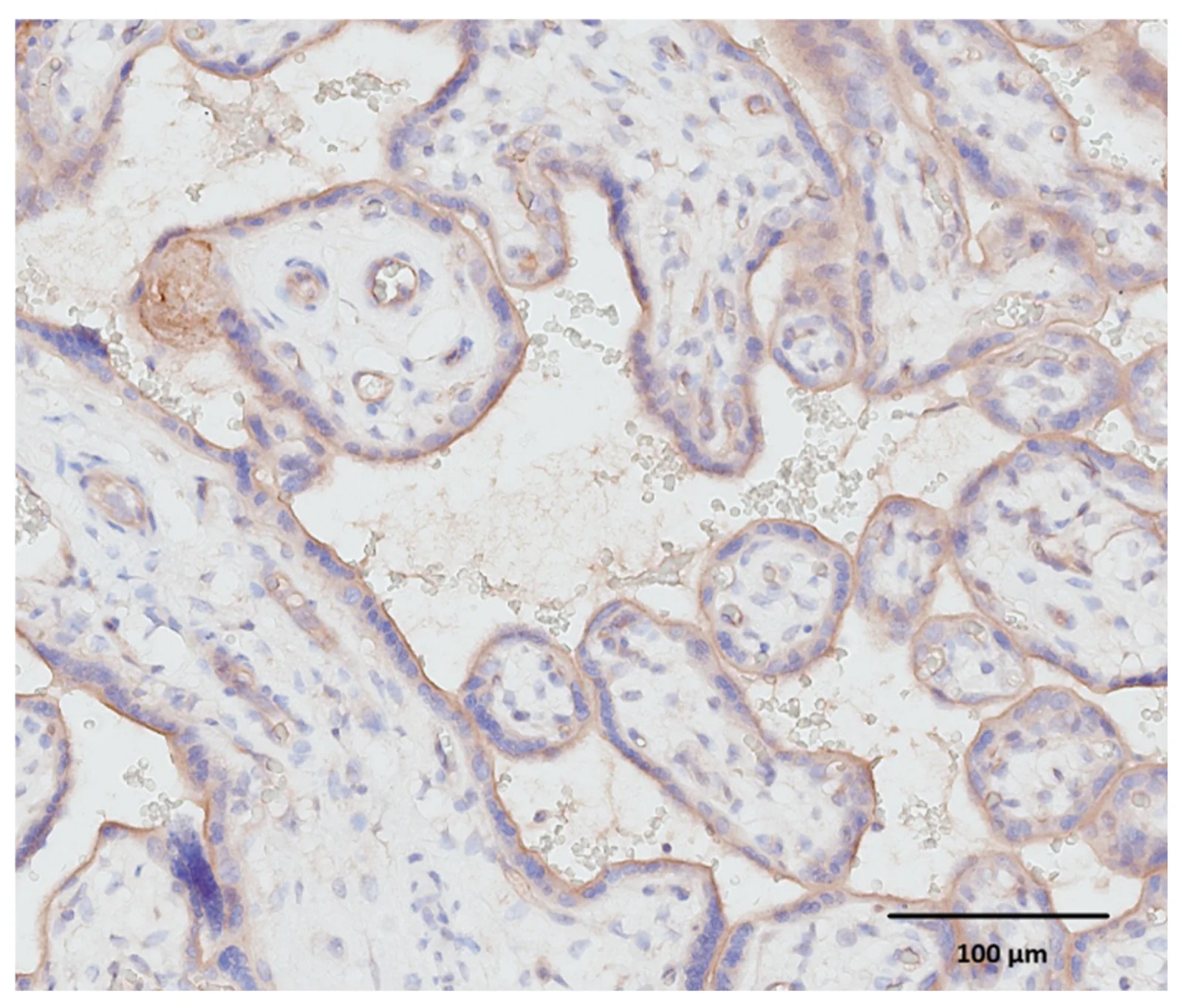
Positive control: placental tissue with strong VEGF-A immunoreactivity in trophoblasts. Scale bar = 100 µm.

VEGF-A staining was present in 71 of 73 cases. Across the full cohort, strong staining occurred in 31 cases (42.5%), moderate staining in 23 (31.5%), weak staining in 17 (23.3%), and absent staining in two (2.7%). Exact intensity agreement was 67/73 (91.8%); linear-weighted κ was 0.913 (percentile-bootstrap 95% CI, 0.835–0.972), ICC(A,1) was 0.963 (95% CI, 0.941–0.976) for percentage positivity, and ICC(A,1) was 0.979 (95% CI, 0.967–0.987) for the modified score. Representative LAMN, SSL, ADCA and HP staining is shown in Figure 2, Figure 3, Figure 4, Figure 5 and Figure 6. Representative placental tissue used as the positive control is shown in Figure 7.

Predominant staining intensity did not differ significantly among diagnostic groups (H = 4.59, permutation *p* = 0.209, epsilon-squared = 0.023). The percentage of positive cells was also not different (H = 6.02, permutation *p* = 0.108, epsilon-squared = 0.044).

**Figure 8:**
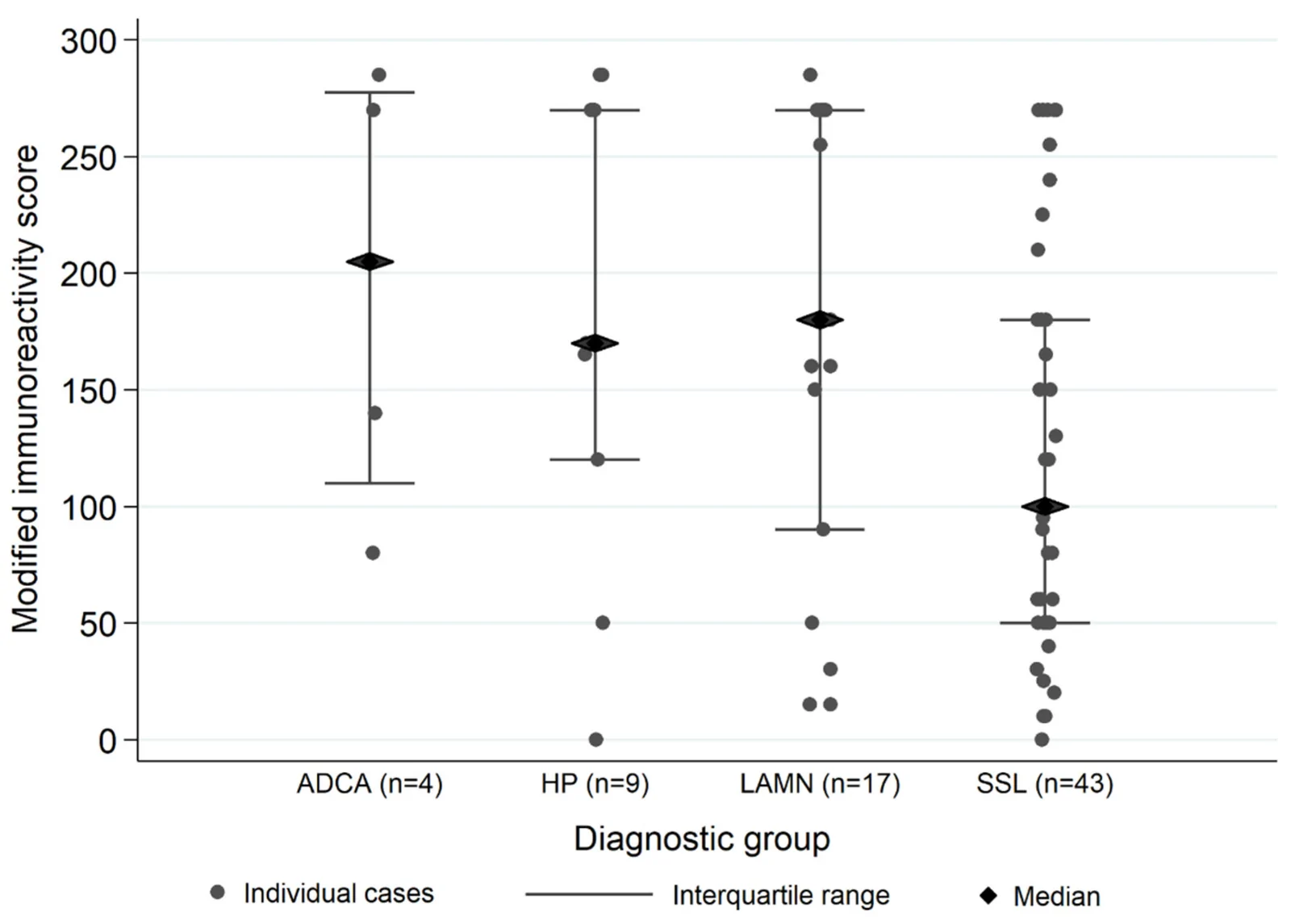
Individual modified immunoreactivity scores by diagnostic group. Filled circles represent individual cases, filled diamonds represent medians, and capped vertical bars represent interquartile ranges.

Median modified immunoreactivity scores were 205 (IQR 110–277.5) for adenocarcinoma, 170 (120–270) for hyperplastic polyp, 180 (90–270) for LAMN, and 100 (50–180) for SSL. The overall comparison did not reach statistical significance (H = 6.78, permutation *p* = 0.073, epsilon-squared = 0.055; stratified percentile-bootstrap 95% confidence interval for epsilon-squared, 0.000–0.272). VEGF-A expression did not differ significantly among the examined appendiceal neoplasm types, although certain trends, particularly lower expression in SSL, were noted (Table 2, Figure 8).

Mitotic activity differed across diagnostic groups (H = 15.38, permutation *p* = 0.000590, epsilon-squared = 0.179). Holm-adjusted pairwise permutation rank-sum comparisons showed higher mitotic counts in adenocarcinoma than in hyperplastic polyp (adjusted *p* = 0.0490), LAMN (adjusted *p* = 0.00175), and SSL (adjusted *p* = 0.000360); the remaining pairwise comparisons were inconclusive.

### 3.2. VEGF-A Staining Intensity in Columnar Mucosal Cells According to Histological Characteristics

The distributions of the assessed histological features are summarized in Table 3. Many feature-by-intensity contingency tables were sparse: between 50% and 75% of expected cell counts were below five. Therefore, Monte Carlo exact-type permutation results were used instead of asymptotic chi-square *p*-values. No histological feature was significantly associated with predominant VEGF-A staining intensity after multiplicity correction. The smallest exact-type *p*-value was observed for inflammatory-infiltrate severity (*p* = 0.064), but all intensity-family q-values were 0.639 or greater. Architecture, crypt serration, basal crypt dilatation, crypt and surface nuclear stratification, inflammatory-infiltrate distribution, luminal mucin, vesicular mucin, and nuclear atypia likewise showed no robust association with staining intensity.

These intensity analyses were descriptive and were not used to infer an effect independent of diagnostic subtype, because several histological features are components of the diagnostic definitions themselves.

### 3.3. Modified VEGF-A Immunoreactivity Score According to Histological Characteristics

Across the ten exploratory histological features, no association with the modified immunoreactivity score met the false-discovery-rate threshold. Global permutation *p*-values (and q-values) were 0.389 (0.648) for architecture, 0.568 (0.740) for crypt serration, 0.029 (0.144) for basal crypt dilatation, and 0.592 (0.740) for crypt nuclear stratification. The nominal basal-dilatation result did not survive multiplicity correction. It was also not significant in the diagnosis-adjusted rank-permutation sensitivity analysis (*p* = 0.107; q = 0.358).

For surface nuclear stratification, median scores were 130 for none, 95 for mild, 240 for moderate, and 180 for marked stratification. This non-monotonic pattern did not reach significance in the global test (H = 7.61, *p* = 0.051, Monte Carlo 95% CI 0.0502–0.0511; q = 0.169). Holm-adjusted pairwise permutation rank-sum comparisons were non-significant (smallest adjusted *p* = 0.071), and the diagnosis-adjusted sensitivity analysis was also non-significant (*p* = 0.282, q = 0.524).

Inflammatory-infiltrate severity (*p* = 0.099; q = 0.246), infiltrate distribution (H = 0.604, permutation *p* = 0.753; q = 0.753), luminal mucin (*p* = 0.253; q = 0.506), and vesicular mucin (*p* = 0.744; q = 0.753) were not associated with the modified score.

Nuclear atypia had the smallest unadjusted *p*-value (*p* = 0.007), but the multiplicity-adjusted result narrowly exceeded the prespecified threshold (q = 0.067). After adjustment for diagnostic subtype, the association remained nominal (*p* = 0.019) but not significant after correction across features (q = 0.186); it was therefore not interpreted as an independent association.

### 3.4. Mitotic Activity and Exploratory Histological Associations

The diagnosis-level difference in mitotic activity is reported in Table 4. In diagnosis-adjusted rank-permutation sensitivity analyses, none of the ten histological-feature associations with mitotic activity remained significant after multiplicity correction, and the smallest result was for inflammatory-infiltrate distribution (*p* = 0.016; q = 0.165). This attenuation indicates that the pooled architecture and atypia findings largely reflected diagnostic composition rather than evidence for independent histological effects. The modified VEGF-A immunoreactivity score was not significantly correlated with mitotic activity (Spearman ρ = 0.203, *p* = 0.085). Accordingly, the mitotic analyses were retained as contextual descriptive findings and were not used to infer a proliferative effect of VEGF-A.

### 3.5. Acute Versus Incidental Presentation

VEGF-A measures did not differ between acute (n = 36) and incidental (n = 37) presentations: predominant intensity *p* = 0.209, percentage positive *p* = 0.91, modified score *p* = 0.421, and mitotic activity *p* = 0.497. Patients with acute presentations were younger (median 64 years, IQR 48.5–74.5) than those with incidental presentations (median 71 years, IQR 65–80; *p* = 0.006). Diagnostic composition showed a borderline imbalance (fixed-margin permutation *p* = 0.052), and all four adenocarcinomas were acute presentations. In a rank-permutation model adjusted for diagnosis and age, presentation remained unrelated to the modified score (*p* = 0.703).

## 4. Discussion

The principal finding of this exploratory study is that VEGF-A immunoreactivity was common across localized appendiceal epithelial lesions, but staining intensity, percentage of positive cells, and the modified immunoreactivity score showed no conclusive differences among diagnostic subtypes. The diagnostic-group effect sizes were small, and the apparent lower median score in SSL was not sufficient to support a subtype-specific interpretation.

The score used here is the product of predominant intensity and percentage of positive cells. It is useful as a transparent semiquantitative summary but differs from the conventional weighted H-score. Reporting it on a 0–300 scale corrects the scale presentation without changing the ordering of cases or the inferential results.

The two closest appendiceal studies addressed advanced disease rather than the localized spectrum examined here. Logan-Collins et al. reported an association between VEGF immunoreactivity and survival in patients with peritoneal surface metastases, whereas Jimenez et al. examined VEGFR-2 expression in appendiceal carcinomatosis [15,16]. Differences in analyte, stage, tissue context, and outcome prevent direct comparison and do not allow the present results to be interpreted as prognostic evidence.

Colorectal studies provide evidence that VEGF-related signaling is detectable in epithelial lesions [17,18], but hyperplastic polyps in this study were neoplastic or polypoid comparators rather than normal controls. Technical positive and negative controls establish staining validity; they do not determine whether expression is increased relative to non-neoplastic appendiceal mucosa. Consequently, the similarity between hyperplastic polyps and other lesions cannot establish that all subtypes have biologically high expression.

None of the ten histological-feature associations with the modified score remained significant after false-discovery-rate correction. Surface nuclear stratification was particularly important to reassess because the medians were non-monotonic, the global *p*-value was 0.051, all Holm-adjusted pairwise comparisons were non-significant, and neither within-subtype nor diagnosis-adjusted analyses supported an association.

Nuclear atypia showed the strongest unadjusted association with the modified score (*p* = 0.007; q = 0.067), while the diagnosis-adjusted result was *p* = 0.019 and q = 0.186. Marked atypia occurred in three adenocarcinomas and moderate atypia in the fourth, which makes diagnostic confounding unavoidable in such a small group. Basal crypt dilatation was nominally associated with the modified score before correction (*p* = 0.029; q = 0.144), but not after adjustment for diagnosis (*p* = 0.107; q = 0.358). These results are best regarded as hypotheses for larger, stratified studies.

Mitotic activity differed by diagnosis, with adenocarcinoma showing higher counts than HP, LAMN and SSL. However, pooled associations between mitotic activity and architecture or atypia were attenuated after accounting for diagnosis, and the correlation between mitotic activity and the modified VEGF-A score did not reach significance. Thus, the mitotic findings support expected biological differences among lesion types but do not demonstrate a link between VEGF-A and proliferation.

Inflammation is a plausible source of VEGF-A through hypoxia- and cytokine-related pathways [13]. Nevertheless, neither inflammatory-infiltrate severity nor distribution showed a robust association with VEGF-A measures, and acute versus incidental presentation was unrelated to VEGF-A after adjustment for diagnosis and age (the acute group was younger). Residual inflammatory confounding cannot be excluded without non-inflamed, non-neoplastic appendiceal controls.

Taken together, the study provides a descriptive baseline rather than evidence for clinical utility. The frequent staining across lesion types may justify further investigation of angiogenic biology, but it does not identify a diagnostic cutoff, a prognostic subgroup, or a rationale for anti-VEGF therapy. Future work should prespecify a primary endpoint, include normal appendiceal mucosa, use standardized quantitative scoring, balance diagnostic strata, and incorporate stage and longitudinal outcomes.

## 5. Limitations

This study has several limitations. Its retrospective, single-center design and use of archival FFPE tissue introduce selection bias and limit control over fixation, processing, and tissue preservation. The cohort represents available rare lesions rather than a prospectively powered sample.

The overall sample was small and unevenly distributed, particularly for adenocarcinoma (n = 4) and hyperplastic polyp (n = 9). Sparse feature categories reduced precision, limited reliable within-subtype testing, and increased susceptibility to chance findings despite permutation methods and multiplicity correction.

No non-neoplastic appendiceal mucosa was included as a biological comparator. Placental and reagent controls verified the technical staining procedure, while adenocarcinoma cases provided only a biological face-validity check. The study therefore cannot determine whether any lesion category has increased expression relative to normal appendix.

Microvascular density was not assessed, precluding evaluation of the relationship between VEGF-A expression and tumor-associated vascularization.

Although interobserver agreement was high, the scoring remained semiquantitative and did not capture the full intralesional heterogeneity that would be represented by a conventional weighted H-score. VEGF-A was evaluated by one immunohistochemical method without molecular confirmation, and no recurrence, progression, treatment-response, or survival data were available. Finally, several histological features overlap with diagnostic definitions, so residual confounding by subtype remains possible.

## 6. Conclusions

VEGF-A immunoreactivity was frequently observed across the spectrum of localized appendiceal epithelial neoplasms examined in this study, with predominantly moderate-to-strong staining. No statistically significant evidence of subtype-specific differences was identified; however, the small and uneven diagnostic groups preclude conclusions of equivalence. Similarly, the overall VEGF-A immunoreactivity score did not differ significantly among adenocarcinoma, hyperplastic polyps, LAMNs, and sessile serrated lesions, although adenocarcinoma showed the highest median immunoreactivity score and sessile serrated lesions the lowest. Most evaluated architectural, mucin-related, and inflammatory features were likewise not consistently related to VEGF-A expression. Basal crypt dilatation and surface nuclear stratification were not associated with VEGF-A after multiplicity correction, pairwise testing or diagnosis adjustment. Taken together, these findings support VEGF-A expression as a common feature of localized appendiceal epithelial neoplasia rather than a marker clearly linked to a specific histological phenotype. The observed associations should be regarded as exploratory, particularly given the small and unevenly distributed diagnostic and histological subgroups. Larger studies are needed to determine whether VEGF-A has a meaningful role in appendiceal tumor biology and progression.

## Figures and Tables

**Table 1:** Baseline characteristics by appendiceal epithelial lesion subtype.

	Adenocarcinoma	HP	LAMN	SSL
number (N)	4	9	17	43
age median (IQR)	66.5 (58.0–68.5)	78.0 (64.0–80.0)	72.0 (63.0–78.0)	69.0 (60.0–79.0)
male, n (%)	0 (0.0)	4 (44.4)	8 (47.1)	24 (55.8)
female, n (%)	4 (100.0)	5 (55.6)	9 (52.9)	19 (44.2)
acute presentation, n (%)	4 (100.0)	2 (22.2)	10 (58.8)	20 (46.5)
incidental presentation, n (%)	0 (0.0)	7 (77.8)	7 (41.2)	23 (53.5)

HP—hyperplastic polyp; LAMN—low-grade mucinous neoplasm of the appendix; SSL—sessile serrated lesion; IQR—interquartile range; N—number of cases.

**Table 2:** VEGF-A immunohistochemical characteristics across appendiceal neoplasm types.

	Adenocarcinoma	HP	LAMN	SSL	*p* *
Staining intensity					0.209
Median (IQR)	2.5 (1.5–3.0)	3.0 (2.0–3.0)	3.0 (2.0–3.0)	2.0 (1.0–3.0)	
Mean	2.25	2.33	2.41	1.98	
Percentage of stained cells					0.108
Median (%) (IQR);	85 (75.0–92.5);	85 (50.0–90.0);	80 (50.0–90.0);	50 (30.0–80.0);	
Mean (%) ± SD	83.8 ± 11.1	66.7 ± 32.8	64.7 ± 28.8	55.0 ± 26.7	
Modified immunoreactivity score					0.073
(0–300)	205 (110–277.5)	170 (120–270)	180 (90–270)	100 (50–180)	
Median (IQR)					
Mean ± SD	194 ± 100	179 ± 107	172 ± 100	119 ± 84	

HP—hyperplastic polyp; LAMN—low-grade mucinous neoplasm of the appendix; SSL—sessile serrated lesion; IQR—interquartile range; SD—standard deviation; *—Monte Carlo permutation *p*-values based on the tie-corrected Kruskal–Wallis H statistic.

**Table 3:** Histopathological features by appendiceal epithelial lesion subtype.

		ADCA (n)	HP (n)	LAMN (n)	SSL (n)
Overall architecture distortion	Slight	0 (0.0%)	8 (88.9%)	11 (64.7%)	41 (95.3%)
	Moderate	0 (0.0%)	1 (11.1%)	6 (35.3%)	2 (4.7%)
	Marked	4 (100.0%)	0 (0.0%)	0 (0.0%)	0 (0.0%)
Serration of the crypts	None	2 (50.0%)	0 (0.0%)	5 (29.4%)	0 (0.0%)
	Mild	0 (0.0%)	7 (77.8%)	6 (35.3%)	18 (41.9%)
	Moderate	2 (50.0%)	2 (22.2%)	6 (35.3%)	25 (58.1%)
Basal dilatation of the crypts	None	3 (75.0%)	8 (88.9%)	11 (64.7%)	10 (23.3%)
	Mild	0 (0.0%)	1 (11.1%)	6 (35.3%)	25 (58.1%)
	Moderate	1 (25.0%)	0 (0.0%)	0 (0.0%)	8 (18.6%)
Nuclear stratification in the crypts	None	0 (0.0%)	4 (44.4%)	4 (23.5%)	11 (25.6%)
	Mild	0 (0.0%)	4 (44.4%)	6 (35.3%)	22 (51.2%)
	Moderate	1 (25.0%)	1 (11.1%)	7 (41.2%)	10 (23.3%)
	Marked	3 (75.0%)	0 (0.0%)	0 (0.0%)	0 (0.0%)
Nuclear stratification on the surface	None	0 (0.0%)	6 (66.7%)	0 (0.0%)	15 (34.9%)
	Mild	0 (0.0%)	2 (22.2%)	4 (23.5%)	22 (51.2%)
	Moderate	1 (25.0%)	1 (11.1%)	11 (64.7%)	6 (14.0%)
	Marked	3 (75.0%)	0 (0.0%)	2 (11.8%)	0 (0.0%)
Inflammatory-infiltrate severity	None	0 (0.0%)	1 (11.1%)	8 (47.1%)	5 (11.6%)
	Mild	0 (0.0%)	2 (22.2%)	5 (29.4%)	11 (25.6%)
	Moderate	1 (25.0%)	6 (66.7%)	4 (23.5%)	16 (37.2%)
	Severe	3 (75.0%)	0 (0.0%)	0 (0.0%)	11 (25.6%)
Inflammatory-infiltrate distribution	Diffuse	4 (100.0%)	6 (66.7%)	3 (17.6%)	19 (44.2%)
	Focal	0 (0.0%)	1 (11.1%)	0 (0.0%)	2 (4.7%)
	Interstitial	0 (0.0%)	1 (11.1%)	6 (35.3%)	17 (39.5%)
	Not applicable	0 (0.0%)	1 (11.1%)	8 (47.1%)	5 (11.6%)
Vesicular mucin	None	3 (75.0%)	1 (11.1%)	3 (17.6%)	6 (14.0%)
	Mild	1 (25.0%)	5 (55.6%)	4 (23.5%)	27 (62.8%)
	Moderate	0 (0.0%)	3 (33.3%)	9 (52.9%)	9 (20.9%)
	Severe	0 (0.0%)	0 (0.0%)	1 (5.9%)	1 (2.3%)
Luminal mucin	None	3 (75.0%)	4 (44.4%)	1 (5.9%)	11 (25.6%)
	Mild	0 (0.0%)	3 (33.3%)	4 (23.5%)	10 (23.3%)
	Moderate	1 (25.0%)	2 (22.2%)	2 (11.8%)	20 (46.5%)
	Severe	0 (0.0%)	0 (0.0%)	10 (58.8%)	2 (4.7%)
Nuclear atypia	None	0 (0.0%)	7 (77.8%)	1 (5.9%)	28 (65.1%)
	Mild	0 (0.0%)	2 (22.2%)	14 (82.4%)	14 (32.6%)
	Moderate	1 (25.0%)	0 (0.0%)	2 (11.8%)	0 (0.0%)
	Severe	3 (75.0%)	0 (0.0%)	0 (0.0%)	0 (0.0%)

ADCA—adenocarcinoma, HP—hyperplastic polyp, LAMN—low-grade mucinous neoplasm of the appendix, SSL—sessile serrated lesion.

**Table 4:** Mitotic activity by appendiceal epithelial lesion subtype.

	Adenocarcinoma	HP	LAMN	SSL	*p* *
Mitotic activity (mitoses per mm^2^)					
Median (IQR)	11 (10–12)	3 (2–5)	1 (1–2)	3 (1–5)	0.00059

Mitotic activity is expressed as the number of mitoses per mm^2^ assessed at ×400 magnification; *—Monte Carlo permutation *p*-value based on the tie-corrected Kruskal–Wallis H statistic.

## Data Availability

The original contributions presented in this study are included in the article/Supplementary Material. Further inquiries can be directed to the corresponding author(s). De-identified individual-level data and data dictionary are provided in Supplementary File S2. Pre-consensus and consensus reader scores are provided in Supplementary File S3. Detailed statistical results, including test statistics, unadjusted *p*-values, Benjamini–Hochberg q-values, diagnosis-adjusted model results, and Monte Carlo confidence intervals, are provided in Supplementary File S4.

## Supplementary Materials

The following supporting information can be downloaded at https://www.mdpi.com/article/10.3390/medsci14060684/s1, Supplementary File S1: Immunohistochemistry Staining Protocol; Supplementary File S2: De-identified Individual-Level Data; Supplementary File S3: Pre-consensus and Consensus VEGF-A Immunohistochemical Scores for Interobserver Agreement Analysis; Supplementary File S4: Complete Statistical Analysis Results, Including Permutation Tests, Multiplicity Corrections, and Diagnosis-Adjusted Sensitivity Analyses.

## References

[1] Misdraji J. (2010). Appendiceal mucinous neoplasms: Controversial issues. Arch. Pathol. Lab. Med..

[2] Carr N.J., Cecil T.D., Mohamed F., Sobin L.H., Sugarbaker P.H., González-Moreno S., Taflampas P., Chapman S., Moran B.J., Peritoneal Surface Oncology Group International (2016). A consensus for classification and pathologic reporting of pseudomyxoma peritonei and associated appendiceal neoplasia: The results of the Peritoneal Surface Oncology Group International modified Delphi process. Am. J. Surg. Pathol..

[3] Carr N.J., Bibeau F., Bradley R.F., Dartigues P., Feakins R.M., Geisinger K.R., Gui X., Isaac S., Milione M., Misdraji J. (2017). The histopathological classification, diagnosis and differential diagnosis of mucinous appendiceal neoplasms, appendiceal adenocarcinomas and pseudomyxoma peritonei. Histopathology.

[4] Nagtegaal I.D., Odze R.D., Klimstra D., Paradis V., Rugge M., Schirmacher P., Washington K.M., Carneiro F., Cree I.A., WHO Classification of Tumours Editorial Board (2020). The 2019 WHO classification of tumours of the digestive system. Histopathology.

[5] Marmor S., Portschy P.R., Tuttle T.M., Virnig B.A. (2015). The rise in appendiceal cancer incidence: 2000–2009. J. Gastrointest. Surg..

[6] Tirumani S.H., Fraser-Hill M., Auer R., Shabana W., Walsh C., Lee F., Ryan J.G. (2013). Mucinous neoplasms of the appendix: A current comprehensive clinicopathologic and imaging review. Cancer Imaging.

[7] Folkman J. (1971). Tumor angiogenesis: Therapeutic implications. N. Engl. J. Med..

[8] Carmeliet P., Jain R.K. (2000). Angiogenesis in cancer and other diseases. Nature.

[9] Ferrara N. (2004). Vascular endothelial growth factor: Basic science and clinical progress. Endocr. Rev..

[10] Ferrara N., Gerber H.P., LeCouter J. (2003). The biology of VEGF and its receptors. Nat. Med..

[11] Hicklin D.J., Ellis L.M. (2005). Role of the vascular endothelial growth factor pathway in tumor growth and angiogenesis. J. Clin. Oncol..

[12] Ellis L.M., Hicklin D.J. (2008). VEGF-targeted therapy: Mechanisms of anti-tumour activity. Nat. Rev. Cancer.

[13] Ramakrishnan S., Anand V., Roy S. (2014). Vascular endothelial growth factor signaling in hypoxia and inflammation. J. Neuroimmune Pharmacol..

[14] Voron T., Colussi O., Marcheteau E., Pernot S., Nizard M., Pointet A.-L., Latreche S., Bergaya S., Benhamouda N., Tanchot C. (2015). VEGF-A modulates expression of inhibitory checkpoints on CD8+ T cells in tumors. J. Exp. Med..

[15] Logan-Collins J.M., Lowy A.M., Robinson-Smith T.M., Kumar S., Sussman J.J., James L.E., Ahmad S.A. (2008). VEGF expression predicts survival in patients with peritoneal surface metastases from mucinous adenocarcinoma of the appendix and colon. Ann. Surg. Oncol..

[16] Jimenez W.A., Sardi A., Ledakis P., Nieroda C., Sittig M., Gushchin V. (2013). The role of VEGFR-2 expression in outcomes and survival of patients with peritoneal carcinomatosis from appendiceal cancer. Eur. J. Surg. Oncol..

[17] Zheng S., Han M.Y., Xiao Z.X., Peng J.P., Dong Q. (2003). Clinical significance of vascular endothelial growth factor expression and neovascularization in colorectal carcinoma. World J. Gastroenterol..

[18] Tjalma J.J., Garcia-Allende P.B., Hartmans E., van Scheltinga A.G.T., Ek W.B.-V., Glatz J., Koch M., van Herwaarden Y.J., Bisseling T.M., Nagtegaal I.D. (2016). Molecular fluorescence endoscopy targeting vascular endothelial growth factor A for improved colorectal polyp detection. J. Nucl. Med..

[19] McShane L.M., Altman D.G., Sauerbrei W., Taube S.E., Gion M., Clark G.M., Statistics Subcommittee of the NCI-EORTC Working Group on Cancer Diagnostics (2005). Reporting Recommendations for Tumor Marker Prognostic Studies (REMARK). J. Natl. Cancer Inst..

[20] Torlakovic E., Skovlund E., Snover D.C., Torlakovic G., Nesland J.M. (2003). Morphologic reappraisal of serrated colorectal polyps. Am. J. Surg. Pathol..

[21] Milne K., Barnes R.O., Girardin A., Mawer M.A., Nesslinger N.J., Ng A., Nielsen J.S., Sahota R., Tran E., Webb J.R. (2008). Tumor-infiltrating T cells correlate with NY-ESO-1-specific autoantibodies in ovarian cancer. PLoS ONE.

[22] Al-kaabi M., Alshareeda A., Jerjees D., Muftah A.A., Green A.R., Alsubhi N.H., Nolan C.C., Chan S., Cornford E., Madhusudan S. (2015). Checkpoint kinase1 (CHK1) is an important biomarker in breast cancer having a role in chemotherapy response. Br. J. Cancer.

[23] Kariri Y.A., Alsaleem M., Joseph C., Alsaeed S., Aljohani A., Shiino S., Mohammed O.J., Toss M.S., Green A.R., Rakha E.A. (2021). The prognostic significance of interferon-stimulated gene 15 (ISG15) in invasive breast cancer. Breast Cancer Res. Treat..

