# Supplementary material for: VEGF-A Immunohistochemical Expression Across Appendiceal Epithelial Lesions: A Retrospective Exploratory Study
Source: Med Sci (Basel). 2026 Oct 19;14(6):684. doi: 10.3390/medsci14060684 (PMC13706902; doi:10.3390/medsci14060684)
Supplement: Supplementary file 1 [file medsci-14-00684-s001.zip › Supplementary file 1.pdf]

Supplementary File 1. Immunohistochemistry Staining Protocol

1. Tissue preparation and sectioning

Archived formalin-fixed, paraffin-embedded (FFPE) appendiceal tissue specimens were used for immunohistochemical analysis. Following surgical excision, specimens had been fixed in 10% neutral buffered formalin for 12–24 hours, exceptionally up to 72 hours, and processed according to institutional pathology protocols before paraffin embedding. Representative lesional areas were selected by a gastrointestinal pathologist based on hematoxylin and eosin (H&E)-stained slides.

Tissue sections were cut at 4 µm thickness using a SLEE CUT 5062 microtome (SLEE Medical, Mainz, Germany) and mounted on Superfrost Plus adhesive slides (Thermo Scientific, Waltham, MA, USA). Mounted sections were incubated on a hot plate at 63°C for 30 minutes before immunohistochemical staining.

2. Immunohistochemical staining

Immunohistochemical staining was performed using the BenchMark ULTRA Advanced automated staining system (Ventana, Tucson, AZ, USA), which automates deparaffinization, antigen retrieval, primary antibody incubation, detection, and counterstaining of tissue sections.

VEGF-A expression was detected using a mouse monoclonal anti-VEGFA antibody, clone VG-1 (ab1316; Abcam, Cambridge, UK), at a dilution of 1:500. The primary antibody incubation was performed at 37°C for 60 minutes.

Automated deparaffinization was performed using EZ Prep solution (1:20 dilution) at 72°C. Heat-induced antigen retrieval was conducted using CC1 buffer at 95°C for 52 minutes. Reaction Buffer (1:20 dilution; Tris-based, pH 7.6 ± 0.2) was used for rinsing and maintaining appropriate aqueous conditions during the staining procedure. Liquid Coverslip oil was applied during incubation steps to prevent evaporation.

Antibody detection was performed using the UltraView Universal DAB Detection Kit (Ventana), a biotin-free indirect detection system employing enzyme-labelled secondary antibodies. Immunoreactivity was visualized using 3,3'-diaminobenzidine (DAB) chromogen and hydrogen peroxide substrate. Counterstaining was performed with modified Gill’s hematoxylin for 8 minutes.

Following staining, slides were washed, dehydrated through graded alcohols, and coverslipped using the Ventana HE600 automated coverslipper.

3. Quality control

Placental tissue was included as an external positive tissue control to confirm the expected VEGF-A immunoreactivity. Strong staining in placental trophoblasts was used as the expected positive staining pattern.

Non-lesional stromal tissue present within the study sections was considered an internal negative tissue control when it showed no detectable staining. No separate no-primary-antibody reagent control was performed.

4. Microscopic evaluation and scoring

Digital slide scanning was performed using the Ventana DP 200 digital slide scanner. Microscopic evaluation was conducted using an Olympus BX51 microscope (Olympus, Tokyo, Japan).

The entire lesional area was first reviewed at low magnification to assess the overall distribution of VEGF-A staining. Five non-overlapping representative fields were subsequently assessed across the lesion, avoiding selection based solely on staining strength.

VEGF-A immunoreactivity was defined as brown cytoplasmic staining in neoplastic columnar epithelial cells. Predominant staining intensity was graded as 0 (absent), 1 (weak), 2 (moderate), or 3 (strong). The percentage of positive neoplastic columnar epithelial cells was visually estimated in 5% increments, from 0% to 100%. Manual cell counting was not performed.

Predominant staining intensity was defined as the intensity category representing the greatest proportion of stained lesional epithelial cells across the assessed area, rather than the maximum focal intensity. Stromal and inflammatory cells were excluded from scoring.

The modified immunoreactivity score was calculated by multiplying predominant staining intensity by the percentage of positive neoplastic epithelial cells, yielding a score ranging from 0 to 300. This measure represents a modified immunoreactivity score rather than the conventional weighted H-score, which sums the proportions of cells at different staining intensities.

5. Interobserver assessment

Two experienced gastrointestinal pathologists independently assessed the stained slides while blinded to clinical data and the recorded diagnostic labels. Slides were coded before assessment. Because morphological features were visible on the slides, blinding to the diagnostic label did not prevent recognition of morphological characteristics.

Intensity and percentage positivity were scored independently before joint consensus review. Discrepancies were resolved with consultation of a third pathologist when required. Consensus values were used for the primary statistical analyses. Interobserver agreement was assessed before consensus scoring using linear-weighted Cohen's kappa for ordinal staining intensity and intraclass correlation coefficients, ICC(A,1), for percentage positivity and the modified immunoreactivity score.
